# Cardiovascular Disease

**DOI:** 10.1186/1472-6874-4-S1-S15

**Published:** 2004-08-25

**Authors:** Sherry L Grace, Rick Fry, Angela Cheung, Donna E Stewart

**Affiliations:** 1 University Health Network Women's Health Program, University of Toronto, 657 University Avenue, Toronto, Canada; 2 Centre for Chronic Disease Prevention and Control, Health Canada, 120 Colonnade Rd, Ottawa, Canada; 3 University Health Network Women's Health Program, University of Toronto, 657 University Avenue, Toronto, Canada; 4 University Health Network Women's Health Program, University of Toronto, 657 University Avenue, Toronto, Canada

## Abstract

**Health Issue:**

Cardiovascular disease (CVD) is the leading cause of death in Canadian women and men. In general, women present with a wider range of symptoms, are more likely to delay seeking medial care and are less likely to be investigated and treated with evidence-based medications, angioplasty or coronary artery bypass graft than men.

**Key Findings:**

In 1998, 78,964 Canadians died from CVD, almost half (39,197) were women. Acute myocardial infarction, which increases significantly after menopause, was the leading cause of death among women.

Cardiovascular disease accounted for 21% of all hospital admissions for Canadian women over age 50 in 1999. Admissions to hospital for ischemic heart disease were more frequent for men, but the mean length of hospital stay was longer for women.

Mean blood pressure increases with age in both men and women. After age 65, however, high blood pressure is more common among Canadian women. More than one-third of postmenopausal Canadian women have hypertension.

Diabetes increases the mortality and morbidity associated with CVD in women more than it does in men. Depression also contributes to the incidence and recovery from CVD, particularly for women who experience twice the rate of depression as men.

**Data Gaps and Recommendations:**

CVD needs to be recognized as a woman's health issue given Canadian mortality projections (particularly heart failure). Health professionals should be trained to screen, track, and address CVD risk factors among women, including hypertension, elevated lipid levels, smoking, physical inactivity, depression, diabetes and low socio-economic status.

## Background

Cardiovascular disease (CVD) is a leading cause of death in Canadian women and men[1]. In general, the onset of CVD is approximately 10 years later in women than in men; women present with a wider range of symptoms[2]; and women are less likely to seek medical care and are less likely than men to be investigated and treated for CVD with specific medications, angioplasty or coronary artery bypass graft [3-7]. Sex differences have also been described in CVD risk factors, including cigarette smoking, depression, low income, elevated serum lipids, hypertension, obesity and lack of physical activity[8,9]. Vulnerable subpopulations include Aboriginal women[10,11], South Asian women[12] and women with diabetes mellitus[13].

## Methods

The results of searches of MEDLINE, PsycINFO and Social Science Abstracts published in English from 1990 to 2002 were used to select the articles included in the literature review. Prevalence data were available through self-report in the National Population Health Survey (NPHS) 1998–1999 cycle[14] and the 2000 Canadian Community Health Survey (CCHS)[15]. Vital statistics databases were analyzed to determine mortality by sex and province[16]. Population rates of hospital admission for CVD by sex and province were obtained with the use of databases from the Canadian Institute for Health Information (CIHI)[1]. Data from the NPHS and the CCHS were analyzed to determine the associations of risk factors such as cigarette smoking, leisure-time activity and overweight with self-reported heart disease, as well as to examine vulnerable subgroups according to income, education, ethnicity/culture, social support, marital status and family structure, by sex and province. The results of the Canadian Heart Health Survey[17] were examined to ascertain the prevalence of high serum cholesterol levels and hypertension, and people's knowledge of the major causes of CVD. International comparisons were obtained from Organization for Economic Co-operation and Development (OECD) data[18].

## Results

### Prevalence and Incidence

The Canadian prevalence of CVD is available only through self-reported data from the NPHS or CCHS. When asked if they had CVD, 3.9% of men and 3.5% of women responded affirmatively, the highest proportion being reported by males in the Atlantic provinces[14]. Although the mortality rate for CVD, particularly ischemic heart disease (IHD), is declining, it is unclear whether the incidence is decreasing as well or the decline in mortality simply reflects increased survival[19].

### Mortality Rate

In 1998, there were 78,964 deaths attributable to CVD in Canada, with generally equivalent numbers in men (39,767) and women (39,197)[15]. Acute myocardial infarction (AMI), incidence of which in women increases significantly after menopause and continues to increase with advanced age, was the overall leading cause of death among women.

**Figure 1:**
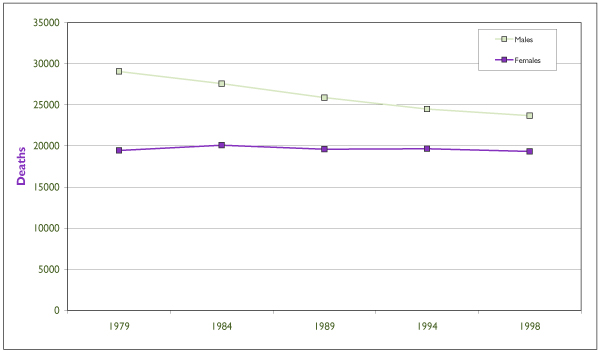
**Ischemic Heart Disease Mortality in Canada, 1979–1998 **Source: Statistics Canada. Vital Statistics, 1999.

Canadian mortality counts for IHD by sex are presented in Figure 1. Regional differences in mortality are more notable for AMI and IHD than for cerebrovascular disease (CBVD). In 1997, rates of mortality from IHD among both men and women were highest in Newfoundland and Labrador; among men they were lowest in Prince Edward Island, and among women they were lowest in British Columbia.

With regard to trends over time, mortality rates declined by half from 1969 to 1997[19]. There is still uncertainty with regard to the causes of this decline, but it is suspected that the reduced incidence is partially explained by declines in risk factors as well as a reduction in case-fatality due to treatment advances. Over the lifespan, Canadian CVD/CBVD mortality rates increase substantially with age, and male rates are considerably higher than female rates for AMI and IHD. Rates of CBVD are similar among men and women until age 55, after which men have increased mortality until age 85, when mortality rates among women become higher.

### Morbidity Rate/Hospitalization

**Figure 2:**
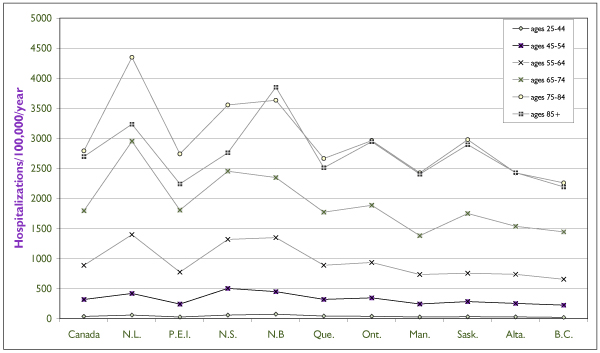
**Hospitalization Rates for IHD Among Women by Province and Age, 1994–1998 **Source: Canadian Institute for Health Information.

Data from the Hospital Morbidity Database of CIHI demonstrate that CVD is the leading cause of hospital admissions for men and women (excluding pregnancy and childbirth)[1]. CVD accounted for 21% of all hospital admissions of Canadian women over the age of 50 in 1999, and rates among older women were higher. Admissions to hospital for IHD were more frequent for men than for women, but the mean length of hospital stay for women surpassed that for men. Figure 2 presents hospitalization rates for IHD among women by age and province. Male rates increased consistently with age, but there was a 10-year delay in AMI among women, purportedly due to the protective effects of estrogen. The decline in morbidity is not as strong as the decline in mortality across time. With regard to provincial variation, Newfoundland and Labrador, Nova Scotia and New Brunswick figureed particularly high rates of IHD.

### International Comparisons

CVD is the leading cause of death worldwide, but rates vary considerably between countries. In countries with established market economies, CVD and CBVD still contribute to approximately half of all deaths in spite of declines in mortality rates over the past 30 years[20]. Overall, CVD mortality rates are about twice as high among men as women, but in many countries the actual number of deaths from CVD among women is similar to that among men because of their longer life expectancy.

**Figure 3:**
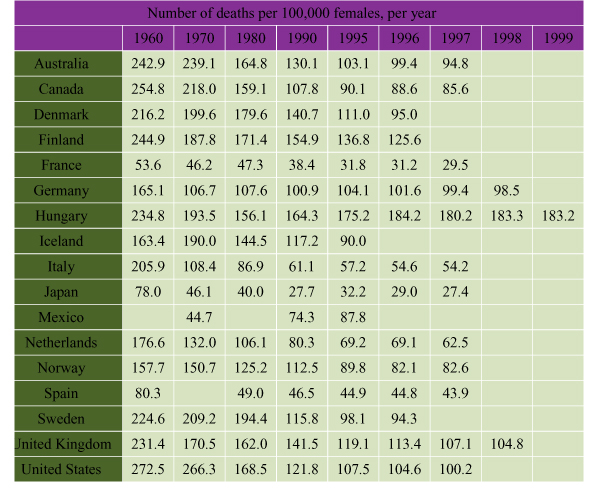
**International Comparisons: IHD Standardized Mortality Rates Among Females, Selected Countries, 1960–1999 **Adapted from: OECD Health Data 2001[18]. Copyright OECD.

Figure 3 displays the IHD mortality rates from 1960 to 1999 for selected countries per 100,000 females[18]. In the 1960s, the highest mortality rates for AMI among women occurred in Australia, New Zealand, Ireland and the United Kingdom (U.K.), while the lowest rates occurred in Japan and Mediterranean countries. By the late 1990s, Canada continued to enjoy lower rates than the United States and the U.K., but rates were considerably higher than those found in Asian countries such as Japan and Korea.

### Comorbidities

#### Hypertension

**Figure 4:**
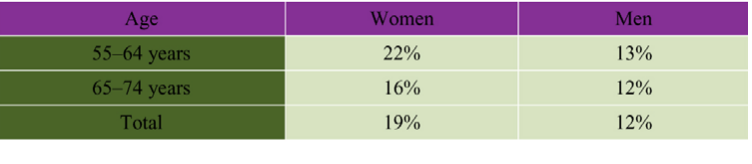
Proportion of Canadians 55 and Older Who Were Aware of TheirHypertension[22]

High blood pressure is an independent risk factor for CVD in women. Mean blood pressure increases with age in both women and men, although after age 65 high blood pressure is more common in Canadian women than Canadian men[21]. Over one-third of post-menopausal Canadian women have hypertension. Women tend to be more aware of the problem than are men and, if the condition is treated, are more likely to have it under control (see Figure 4).

#### Lipid Profile

**Figure 5:**
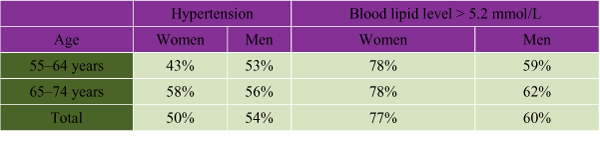
Proportion of Canadians 55 and Older With Hypertension and Elevated Blood Lipid Level** Based on Heart Health Survey Data 1986–1992[23]

High blood cholesterol in women is a major risk factor for CVD, and this is amplified by smoking and hypertension. The prevalence of elevated total lipids in women increases rapidly after menopause, such that by age 55 women have higher levels than men (see Figure 5)[24]. Although high total cholesterol in women does not seem to be as great a risk as it is in men, the combination of low levels of high-density lipoprotein (HDL) and elevated triglycerides increases women's risk of death from CVD tenfold. Forty-three percent of Canadian women aged 18 to 74 have a total blood cholesterol above the recommended threshold of 5.2 mmol/L[25]; 32% of women have elevated low-density lipoprotein levels (> 3.4 mmol/L); and 4% of women have low HDL levels (< 0.9 mmol/L).

#### Diabetes Mellitus

Diabetes mellitus (DM) increases rates of mortality and morbidity from CVD more in women than in men and eliminates the advantage for women in all atherosclerotic disease outcomes except stroke [26-29]. Diabetic women are significantly more likely than diabetic men or non-diabetic women to have coronary events. DM is often associated with obesity, a sedentary lifestyle and lower socio-economic status (SES)[30].

#### Depression

Depression also contributes to the incidence of and poorer recovery from CVD [31-36], particularly for women, who experience twice the rate of depression as men[37]. Beaudet[38] showed that Canadians aged 55 to 74 who had had a depressive episode in the previous 12 months were nearly three times as likely to have CVD within the following four years as people who had not experienced any depressive episode (odds ratio [OR] = 2.7, 95% confidence intervals [CI] 1.01–7.04). Frasure-Smith et al[39]. analyzed the impact of gender and depression after AMI in a Canadian sample and found that 8.3% of the depressed women died of cardiac causes in contrast to 2.7% of the non-depressed. Depression during hospitalization was found to have a significant impact on long-term mortality, with the increased risk being largely independent of CVD severity. Patients of both sexes who experienced depression tended to report more advanced cardiac disease.

### Vulnerable Subgroups

#### Socio-economic Status

According to self-reported data, Canadian women and men with CVD tend to have annual income levels in the range of $5,000 to $30,000[14]. Moreover, most Canadians with self-reported CVD have less than secondary education[14], and those with less education are more likely to show early stage atherosclerosis for any given age group[40]. Socio-economic determinants act in part through an increased prevalence of risk factors, but they also have an independent effect that may be mediated through social isolation, coping styles, health behaviour, job strain or stress, and anger or hostility[41,42].

#### Ethnicity/Culture

Approximately 1 in 5 Canadians is a first-generation immigrant. In addition to genetic factors, immigrants tend to bring with them cultural habits (e.g. food choices, smoking behaviour) that influence their risk of developing CVD/CBVD[43]. The largest non-European migrant groups are from China and South Asia, and these groups show lower all-cause mortality rates among both men and women. However, South Asian immigrant women have the highest rate of IHD among Canadian women[19,44]. Studies from the United States show increased rates of IHD among Black women [45-47]. Canadian data indicate that 7.3% of Black women versus 2.8% of Black men have self-reported CVD, as compared with 3.5% and 3.9% for the entire population respectively[14].

#### Social Support/Family Structure

Social support plays an important role in an individual's ability to maintain a healthy lifestyle and recover from illness and surgery [48-51]. This may be a greater problem for women, many of whom are widowed or isolated[52]. For instance, 6.8% of Canadian men with self-reported CVD versus 3.9% of women are married, and 15.6% of men with self-reported CVD versus 16.5% of women are widowed[14]. Moreover, women with self-reported CVD are more often living on their own (9.7%), whereas men are most frequently living with a partner (11.5%)[14]. These differences in risk factors likely arise from the age-distribution shift in women's CVD.

#### Associations between Risk Factors and Self-Reported Heart Disease by Sex

**Figure 6:**
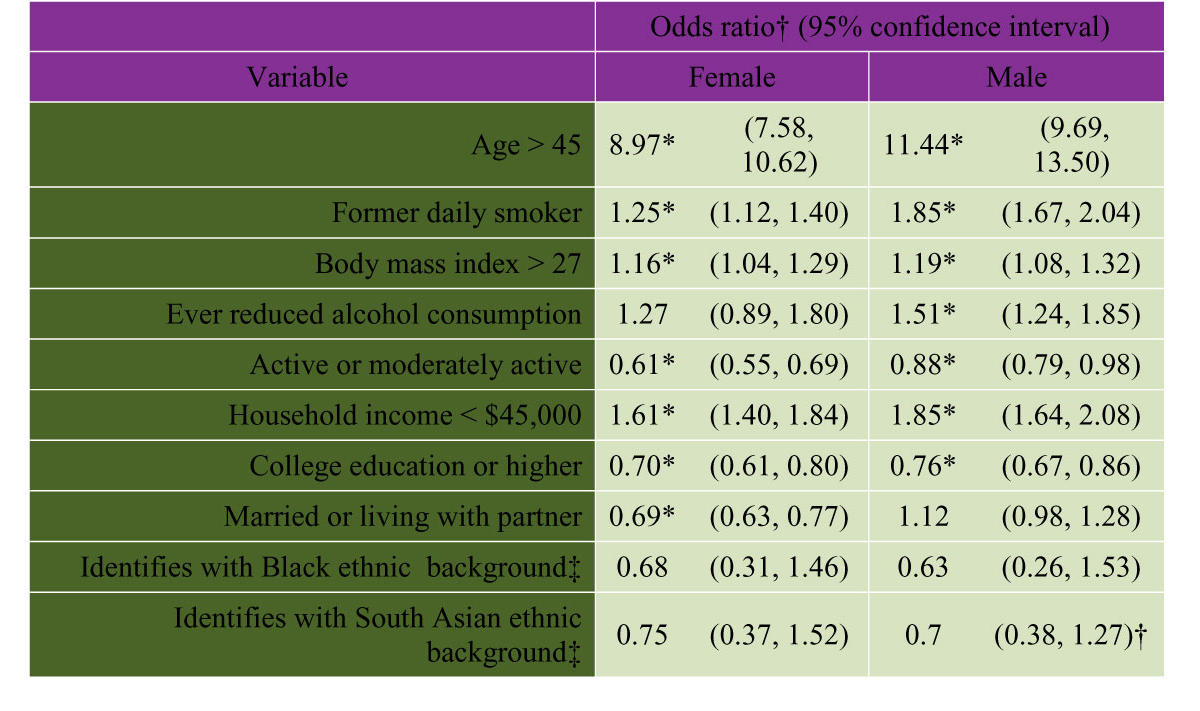
**Bootstrapped Logistic Regression Analysis for Variables Associated With Self-Reported Heart Disease in Canadian Females and Males. **The odds ratio estimates and their associated confidence intervals were calculated using the Statistics Canada bootstrap weights for the CCHS and the SAS macro program, which was written for that purpose. ‡ Black and South Asian ethnic status show odds ratios in the direction of a protective effect for both sexes, but the confidence intervals suggest that these are not statistically robust results. This could be because of a lack of statistical power. Despite the 130,000 respondent records in the CCHS, there are relatively few people represented with these ethnic backgrounds who report currently living with heart disease. Source: 2000 Canadian Community Health Survey (Statistics Canada) (This analysis is based on the Statistics Canada CCHS, Cycle 1.1, 2000. All computations on these data were done by Health Canada and the responsibility for the use and interpretation of these data is entirely that of authors.)

Data from the 2000 CCHS concerning risk factors and vulnerable subgroups were used to examine self-reported heart disease in women and men in a multivariate logistic regression (Some caveats to the use of a cross-sectional survey like the CCHS should be noted. Risk factors such as current daily smoking and current heavy alcohol consumption tend to figure odds ratios that suggest they are protective for heart disease. This is because of a survey bias stemming from the fact that many people engage in these behaviours and do not quit until some related disease has been diagnosed. Their current smoking and drinking are truly associated with lack of a diagnosis. Questions in the NPHS/CCHS surveys are not written in such a way that responses can be used to definitively characterize long-term levels of smoking and drinking. For instance, it is not possible to calculate pack years of smoking from the CCHS data. The two variables "former daily smoker" and "ever reduced alcohol consumption for any reason" are surrogates for past heavy drinking and smoking. They are biased towards a probability of a current diagnosis of heart disease, since many people quit their habits on the advice of a clinician. There is a problem with using a self-reported heart disease outcome, particularly in elderly people, among whom it is by far more common and is greatly under-reported, especially in lower education groups. Caution is warranted when analyzing prevalent cases of coronary heart or other frequently fatal diseases, given that the very high initial mortality may result in the overrepresentation among prevalent cases of people protected from a poor prognosis). (see Figure 6). For both sexes, increasing age, lower household income, former daily smoking, and BMI of less than 27 all showed a positive risk for heart disease, and being physically active and having a higher educational level were protective. However, although being married appears to be protective for females it is neither protective nor a risk for males. This is in line with data presented elsewhere showing that family structure and social support are integral protective factors for women.

### Risk Factors

#### Behavioural

##### Exercise

Physical activity reduces CVD rates of morbidity and mortality among women[53]. The Canadian Heart Health Survey reported that 36% of Canadian women aged 18 to 74 were classified as physically inactive based on their self-report of leisure-time physical activities. In the 1998–1999 NPHS, 53% of Canadian adults were classified as physically inactive, and this was more prevalent among Canadian women (56.9%) than men (48.6%)[14], in populations with lower SES, and with increasing age[54] (please also refer to the "Personal Health Practices" chapter in this report).

##### Smoking

Cigarette smoking is the main preventable CVD risk factor for women and men. It is a stronger risk factor for AMI in middle-aged women than in men, and in women who use oral contraceptives[21]. In 1998–1999, more men than women were daily smokers in all age groups except the under 24 group (21% of women versus 20% of men)[14]. For instance, daily smoking between the ages of 25 and 39 was reported by 30% of men and 28% of women, between the ages of 40 and 54 by 28% of men and 24% of women, and for those aged 55 and over by 18% of men and 13% of women. Smoking rates tend to be higher in Quebec and the Atlantic provinces than in other Canadian provinces (please also refer to the "Sex And Gender Differences in Smoking and Self Reported Indicators of Health in Canadian Women" chapter of this report).

##### Overweight/Obesity

Obesity is highly prevalent among Canadians, and notable increases across North America have been the trend. The Canadian Heart Health Survey[17] reported that 41% of Canadian women aged 18 to 74 years were overweight (defined as a BMI of > 25 kg/m2), and 27% were obese (defined as a BMI > 27 kg/m2). The prevalence of obesity was shown to increase steadily with age and to be higher among men than women (please also refer to the "Physical Activity and Obesity in Canadian Women" chapter of this report).

### Interventions Aimed at Women

#### Prevention

Mortality from CVD and CBVD among Canadian women has generally declined over the past three to four decades[3]. However, given that reduced mortality has been seen to a greater degree among men and those of northwestern European ancestry, we must do more. Unfortunately, there are currently no representative Canadian data concerning the efficacy of primary or secondary CVD prevention programs.

North American data generally show significant sex differences in referral to and participation in secondary prevention programs such as cardiac rehabilitation (CR) [55-60]. In general, 20% fewer women are enrolled in CR than men,[61,62] a proportion significantly lower than expected on the basis of morbidity[63]. Despite women's lower participation[64,65], women of all ages benefit from CR [66-69], with improvements in functional capacity, coronary risk and psychosocial well-being that are comparable with or exceed those of men[66].

#### Diagnosis/Detection Programs

A gap exists in Canadian CVD surveillance data with regard to diagnosis and detection programs. Data from the Canadian Heart Health Survey (1986–1992) show that risk factors for CVD are under-diagnosed and under-detected. For instance, only 42% of Canadians with hypertension were aware that they had hypertension[22]. Of those aged 18 to 74, 26% of men and 18% of women were hypertensive. Among men, 47% were unaware of their hypertensive state, for 21% the condition was not treated and was uncontrolled, for 19% it was treated but not controlled, and for 13% it was treated and under control. Among women, 35% were unaware of their hypertensive status, for 15% it was not treated and was uncontrolled, for 29% it was treated but not controlled, and for 20% it was treated and under control[70].

#### Treatment/Interventions

Canadian female AMI patients in every age group are less likely to undergo either percutaneous transluminal coronary angioplasty (PTCA) or coronary artery bypass grafting (CABG) revascularization[1,71]. This may be partially explained by women's higher age at CVD onset, given that the best candidates for revascularization are younger individuals without comorbid conditions.

## Discussion

### Data Limitations

To improve our understanding and management of CVD among women, we must examine surveillance capabilities, research methodologies, and heart health policies and services (see also the gaps identified in the bulleted points below). With regard to the surveillance of the diagnosis and detection of CVD, we urgently need incidence estimators at the population level (such as the MONICA/ICONS project in Nova Scotia). We lack data on recent physical measures (i.e. hypertension, lipid profiles), for which self-reporting is notoriously poor. We need recent data on who is undergoing treatment for hypertension, hyperlipidemia and depression, and the effectiveness of these treatments. We are unable to capture the number of women or men undergoing stress tests, angiography, echocardiography or 24-hour blood pressure monitoring.

Information on risk factor incidence and prevalence across the lifespan is also lacking. Methodologically speaking, person-oriented data for women (and men) would enable us to follow Canadians longitudinally through the health care system and across the lifespan.

Surveillance data regarding health services evaluation are lacking. We are unable to determine the prevalence of medication prescription, compliance with treatment, or prevention of CVD and CBVD. Physician service utilization data for CVD/CBVD (as compared with those without CVD/CBVD), patient access to physician offices for prevention of CVD/CBVD (i.e. determined through physician billing data at the provincial level), and hospitalization data for patients with CVD/CBVD versus those without it are deficient. In short, the following gaps are notable:

• incidence indicators at the population level;

• recent data on physical measures, such as hypertension and lipid profile;

• information on people undergoing treatment for hypertension and hyperlipidemia, and the control rate;

• person-oriented data to follow people through the health care system;

• prevalence of prevention and detection programs, including community heart health and smoking cessation programs;

• national drug data for the treatment and prevention of CVD/CBVD;

• the changing prevalence of congestive heart failure; and

• the number of women and men undergoing stress tests, angiograms, echocardiography and holteronitoring.

### Policy Considerations

With regard to healthy public policy, CVD needs to be recognized as a women's health issue, given the Canadian mortality projections, the aging population, and rampant inequities in health care access and provision. Health professionals should be trained to screen and address CVD risk factors in women, such as hypertension, elevated lipid levels, smoking, physical inactivity, depression, diabetes mellitus and low SES. We need to continue developing and evaluating educational resources for women across the lifespan regarding their risk for CVD and symptom presentation. Efforts to encourage healthy eating habits and physical activity through a multiplicity of approaches should be pursued. This may include working with local governments, workplaces, health care providers and the media to promote the importance of physical activity while recognizing the unique circumstances of women and girls (e.g. by providing a safe environment). Finally, attention must be paid to barriers to physical activity among women of diverse ethnocultural backgrounds and social classes.

## Notes

The views expressed in this report do not necessarily represent the views of the Canadian Population Health Initiative, the Canadian Institute for Health Information or Health Canada.
